# Healthcare unplugged: Disparities in broadband internet and health facility access among US counties

**DOI:** 10.1371/journal.pdig.0000732

**Published:** 2026-07-23

**Authors:** Khushi Kohli, Stephanie Wang, Cody Chou, Bhav Jain, Kavya M. Shah, Mahi Kohli, Edward C. Dee, Sandeep Palakodeti

**Affiliations:** 1 Harvard Medical School, Boston, Massachusetts, United States of America; 2 Columbia University Vagelos College of Physicians and Surgeons, Manhattan, New York, United States of America; 3 Stanford University School of Medicine, Stanford, California, United States of America; 4 Department of Radiation Oncology, Memorial Sloan Kettering Cancer Center, New York, New York, United States of America; 5 DSP Health, Granville, Ohio, United States of America; McGill University, CANADA

## Abstract

Amid the COVID-19 pandemic, telemedicine emerged as a promising solution for early disease detection. However, access may remain limited in communities with reduced access to outpatient health facilities key for timely screening. Here, we correspond broadband internet access with access to health facilities and elucidate rural-urban disparities. For 3,133 US counties, we extracted data on the population density of outpatient care centers, diagnostic labs, and nursing/residential care (2017 National Neighborhood Data Archive), broadband access stratified based on the median percentage (9.8%) of households without broadband (2022 FCC Mapping Broadband Health in America), urbanicity level, and sociodemographic covariates (2022 American Community Survey). Two-sample t-tests substantiated the association between broadband access and facility densities. Multivariable linear regressions quantified the association between broadband access and facility densities, adjusting for sociodemographic covariates, and geographical heatmaps visualized trends in broadband access, urbanicity level, poverty rate, and facility densities. Heatmaps revealed that regions with limited healthcare facilities—particularly the Great Plains and Southwest—overlap with areas of low broadband coverage. Compared to counties with high broadband access, those with low access had fewer outpatient care centers (mean 10.46 vs. 11.91 per 100,000 residents, P < 0.001) and diagnostic labs (mean 1.91 vs. 3.95 per 100,000 residents, P < 0.001). They also demonstrated higher poverty rates, older populations, increased rurality, and lower educational attainment, employment, and insurance (all P < 0.001). Multivariable regression confirmed that declining broadband access corresponds with lower densities of outpatient care centers (β = −0.045), diagnostic laboratories (β = −0.024), and nursing/residential care facilities (β = −0.089; all *P* < 0.001). Ultimately, US communities with the fewest physical health resources—particularly in rural, low-income counties—are also less likely to have broadband access. Addressing this inequity necessitates coordinated investment in broadband expansion and targeted health services deployment.

## Author summary

Rural and low-income populations face significant barriers to accessing healthcare, leading to later diagnoses and worse outcomes. Telemedicine has often been proposed as a tool to bridge these disparities—however, many telehealth services rely on broadband internet, which is not equally accessible across regions and socioeconomic groups. In this study, we analyzed over 3,000 US counties to elucidate whether communities with low broadband access also house fewer brick-and-mortar healthcare facilities—including outpatient care centers, diagnostic labs, and nursing or residential care facilities. We found that counties with low broadband access are not only more rural, older, and socioeconomically disadvantaged, but they also house significantly fewer outpatient care centers and diagnostic labs. With limited access to both preventative in-person healthcare and broadband-enabled telehealth services, these counties face a “dual disparity” that disproportionately leaves behind communities already burdened by social vulnerability.

Building on these findings, we highlight the need for policies that increase the availability of broadband infrastructure, healthcare personnel, and in-person healthcare care, bringing much-needed care into communities facing these dual disparities. Through this targeted approach, we can ensure that the full functionalities of telehealth services can reach the United States’s rural, low-income communities facing the highest burdens of disease.

## Introduction

With the recent and rapid growth of telemedicine services in the United States, disparate access to broadband internet poses major health challenges, particularly in rural communities [1]. Rural America already faces formidable healthcare inequities — while 25% of Americans live in rural communities, only 10% of physicians practice there [2]. Consistent trends of frequent hospital closures, sparser healthcare facilities, and lack of insurance coverage further exacerbate healthcare inaccessibility in rural communities, associated with higher burdens of diseases such as obesity, diabetes, and cancer [3,4]. Telemedicine serves as a potentially promising solution to overcome the inadequacies of physical health facilities in rural areas by circumventing the need to commute long distances to access healthcare.

Improving healthcare outcomes in these regions relies heavily on telemedicine. By allowing patients to consult with healthcare providers remotely, telemedicine can reduce the need for travel thus saving time and money and making it easier for patients to receive timely care [5]. It can also provide access to specialists who might not be available locally. For chronic disease management, telemedicine can enable continuous monitoring and follow-up care. Regular check-ins via telehealth and remote monitoring help in identifying early symptoms and managing conditions like diabetes, hypertension, and cardiovascular diseases effectively, thereby reducing hospitalizations and emergency visits [6].

Yet, the potential of telehealth to improve healthcare accessibility in rural communities is hindered by the requirement of broadband internet for many of its functionalities, including video visits, remote monitoring of medical devices, and nonsynchronous messaging [7]. Despite a 38-fold increase in telehealth usage post-pandemic, high-burden communities with reduced access to local outpatient health facilities may still face limited telehealth access. Access to broadband internet significantly influences the utilization of telemedicine services. Broadband internet has been expanding across the United States, yet significant gaps remain, particularly in rural and underserved areas. Current barriers include not just the lack of infrastructure but also issues like affordability and digital literacy, which prevent people from making full use of available services [8].

In this study, we focus on three types of health facilities: outpatient care centers, diagnostic labs, and nursing/residential care facilities. These facilities were chosen because they play important roles in providing comprehensive healthcare services. Outpatient care centers offer primary and specialized care, diagnostic labs provide essential testing and screening services, and nursing/residential care facilities support long-term care needs. High broadband access allows these facilities to integrate telehealth strategies and solutions effectively. For example, outpatient care centers can conduct virtual consultations, reducing the need for in-person visits. Diagnostic labs can use telehealth to provide remote consultations and quick access to test results, improving the speed and accuracy of diagnoses. Nursing and residential care facilities can leverage telehealth for remote monitoring and consultations, ensuring continuous care without the need for frequent hospital visits [9].

We hypothesize that regions with lower access to these facilities may also have lower access to broadband internet. If true, such communities would experience “dual disparities” in access to healthcare, facing barriers in accessing not only physical health services but also the benefits of telemedicine. This could lead to delayed diagnoses, poor management of chronic conditions, and ultimately worse health outcomes in these communities.

Thus, this study aims to i) correlate broadband internet access with the availability of various health facilities and ii) highlight rural-urban and income-based disparities in broadband access. By addressing these issues, we hope to underline the importance of targeted interventions that can bridge the gap in both internet connectivity and healthcare services, ultimately improving health outcomes for rural and low-income populations.

## Methods

### Data collection

#### Population density of health facilities.

Using the University of Michigan’s 2017 National Neighborhood Data Archive (NaNDA), we extracted data on the population density of outpatient care centers, diagnostic laboratories, and nursing/residential care facilities for 3,133 US counties [10]. In this database, outpatient care centers are defined to include substance abuse treatment centers, kidney dialysis centers, and community health clinics; diagnostic laboratories include facilities that provide services related to medical testing and imaging; nursing/residential care facilities include skilled nursing facilities, inpatient care hospices, assisted living facilities that provide full-time nursing care, and facilities offering residential intellectual and developmental disability care. However, facilities that primarily offer housekeeping and social services were excluded from the definition of nursing/residential care facilities. The densities of these facilities were reported per 1,000 residents in the NaNDA database and were subsequently converted to densities per 100,000 residents.

#### Broadband and demographic data.

We collected county-level data on broadband access (stratified based on the median percentage of households without broadband) from the FCC’s 2020 Mapping Broadband Health in America Platform [11]. This data was derived from the FCC’s February 2020 filings of Form 477 by internet service providers, which includes information on national broadband speed and coverage rates. We then stratified counties into two groups: a “high broadband access” group, in which the percentage of households without broadband was below the median threshold of 9.8%, and a “low broadband access” group, in which the percentage of households without broadband access was above the median threshold of 9.8%.

We collected county-level data on broadband access (stratified based on the median percentage of households without broadband) from the FCC’s 2020 Mapping Broadband Health in America Platform. We also collected 5-year estimates of county-level urban-rural status, percentage of households experiencing poverty, employment and insurance rate, sex, age, and race (stratified by percentage of participants identifying as White, Black, Asian, American Indian or Alaska Native, Native Hawaiian or Pacific Islander, or Other) from the 2021 American Community Survey (ACS). The ACS defined urbanicity level using 2013 National Center for Health Statistics (NHCS) Urban-Rural Classification Scheme, which bins counties into one of six categories (1 - Large Central Metro, 2 - Large Fringe Metro, 3 - Medium Metro, 4 - Small Metro, 5 - Micropolitan, 6 - Noncore). A household was designated as living below the poverty line if their total annual income fell below the US Census Bureau’s American Community Survey, which accounts for family size and number of children. Individuals aged 16 and above were classified as employed if they were “at work” or “with a job but not at work” during the week before the respondent answered the survey. Individuals with any of the following forms of health insurance were classified as “insured”: Employer-based, Directly-purchased, Medicare, Medicaid/Medical Assistance, TRICARE, VA health care, Indian Health Service, and Other [12].

**Fig 1:**
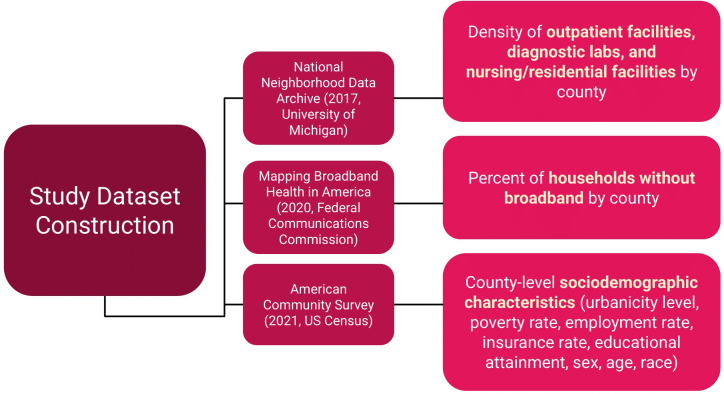
Study dataset construction. Schematic illustrating the construction of the analytical dataset, linking broadband access, sociodemographic characteristics, and access to health facilities at the county level.

Although our study integrates data sources with varying reference years, this approach was carefully selected to balance data availability with geographic coverage. The ACS 5-year estimates (2017-2021) span the full time range of the two other data sources (2017 NaNDA health facility data; 2020 FCC broadband access data), and were selected over 1-year estimates to account for short-term fluctuations in demographic data—particularly in small-population and rural counties. The structural health facility distribution data, obtained from the 2017 NaNDA dataset, are generally stable and unlikely to drastically shift at the county level within short time periods. Together, these sources offer a contemporaneous and appropriate snapshot of county-level broadband access, demographic context, and healthcare infrastructure in the US (**Fig 1**).

### Mapping

To visualize geographical trends in healthcare facility density, broadband access, and poverty rates across the country, we used the QGIS 3.36.1 geomapping software [13]. Specifically, we visualized i) the population density per 100,000 residents of outpatient care centers, diagnostic laboratories, and nursing/residential care facilities, ii) the percentage of households without broadband access, and iii) the percentage of households below the poverty line to the US counties on a base map obtained from the US Census Bureau’s TIGER/Line Shapefiles from 2020 (to align with the reference year of the FCC’s 2020 Mapping Broadband Health in America Platform data), which are in the public domain [14]. We then created geographical heatmaps for each variable using the mapped dataset. We have also included a supplementary figure depicting commonly referenced geographical regions of the United States, which will be referenced in the interpretation of geospatial analyses throughout the manuscript (**S1 Fig**).

### Data analysis

Using two-sample t-tests, we substantiated the association between “high” and “low” broadband access stratified by the national county-level median percentage of households without broadband (9.8%) and the population density of outpatient care centers, diagnostic laboratories, and nursing/residential care facilities, respectively. A sensitivity analysis using more stringent definitions of “high” and “low” broadband access was also performed to assess the robustness of these findings. For the sensitivity analysis, we defined counties with “high broadband access” as those within the lowest quartile nationally for the percentage of households without broadband access (≤ 2.3%). We defined counties with “low broadband access” as those within the highest quartile nationally for the percentage of households without broadband access (≥ 26.8%). Moreover, we conducted unadjusted and adjusted multivariable linear regressions to quantify the association between i) broadband internet access and the density of each facility and ii) household poverty rate and the density of each facility, adjusting for all sociodemographic covariates described above. All data analysis was conducted in R Version 4.3.2 [15].

## Results

### Baseline descriptive analysis

Two-sample t-tests revealed that US counties with reduced broadband access are significantly more likely to face reduced availability of various in-person health facilities. Counties with low broadband access had significantly fewer outpatient care centers (mean 10.46 vs. 11.91 per 100,000 residents, P < 0.001) and diagnostic labs (mean 1.91 vs. 3.95 per 100,000 residents, P < 0.001) compared with counties with high broadband access. However, broadband access did not show a statistically significant association with the density of nursing and residential care facilities (mean 31.36 vs. 30.66 facilities, P = 0.39) (**Table 1**).

**Table 1:** US county-level baseline characteristics and health facility density per 100,000 residents, stratified by access to broadband internet (two-sided *t*-test, comparing counties with low vs. high broadband access). Counties with high broadband access were defined as those in which the percentage of households without broadband access was below the national county-level median (9.8%), and counties with low broadband access were defined as those at or above the national median. Adults identifying as “Other Race” include individuals identifying as Native Hawaiian or Other Pacific Islander or any other race not otherwise specified, including individuals identifying as multiracial.

	Density (per 100,000 residents)			*P* value (two-sided *t* test, comparing counties with low vs. high broadband access)
	All US Counties	US counties with low broadband access	US counties with high broadband access	
**NHCS Urban-Rural Code**	4.64	5.20	4.08	*P* < 0.001
**Household Poverty Rate**	10.17	11.31	9.03	P < 0.001
**Age**	41.60	42.50	40.70	P < 0.001
**Percent of Adults Who Are Male**	50.46	50.90	50.02	P < 0.001
**Percent of Population Identifying as White**	79.05	78.65	79.46	0.19
**Percent of Population Identifying as Black**	8.87	10.13	7.60	P < 0.001
**Percent of Population Identifying as Asian**	1.43	0.71	2.15	P < 0.001
**Percent of Population Identifying as American Indian or Alaska Native**	1.90	2.58	1.20	P < 0.001
**Percent of Population Identifying as Other Race**	2.81	2.46	3.16	P < 0.001
**Percent of Adults With a High School Degree**	88.34	87.27	89.41	P < 0.001
**Percent of Adults Who are Employed**	55.12	52.60	57.65	P < 0.001
**Percent of Adults With Insurance**	90.48	89.63	91.33	P < 0.001
**Outpatient Care Centers**	11.18	10.46	11.91	*P* < 0.001
**Diagnostic Labs**	2.93	1.91	3.95	*P* < 0.001
**Nursing/Residential Facilities**	31.01	31.35	30.66	*P* = 0.39

In addition to experiencing reduced availability of in-person health facilities, US counties with low broadband access also appear to experience elevated indicators of social vulnerability. Counties classified as having low broadband access demonstrated higher household poverty rates (mean 11.31% vs. 9.03%; P < 0.001), older populations (mean age 42.5 vs. 40.7 years; P < 0.001), and increased rurality (mean NHCS urban-rural classification code 5.20 vs. 4.08; P < 0.001). These counties were also observed to have slightly higher proportions of male residents (mean 50.90% vs. 50.02%; P < 0.001), lower average levels of educational attainment (87.3% vs. 89.4% of adults with a high school degree; P < 0.001), employment rates (52.6% vs. 57.7%; P < 0.001) and insurance coverage (89.6% vs. 91.3%; P < 0.001).

With respect to racial composition, the percentage of residents identifying as White was similar across both groups (78.7% vs. 79.5%; P = 0.19). However, counties with low broadband access demonstrated a higher percentage of residents identifying as Black (10.1% vs. 7.6%; P < 0.001) and American Indian or Alaska Native (2.6% vs. 1.2%; P < 0.001), but a lower percentage of residents identifying as Asian (0.7% vs. 2.2%; P < 0.001) or Other Race (2.5% vs. 3.2%; P < 0.001 (**Table 1**).

### Mapping analysis

**Fig 2:**
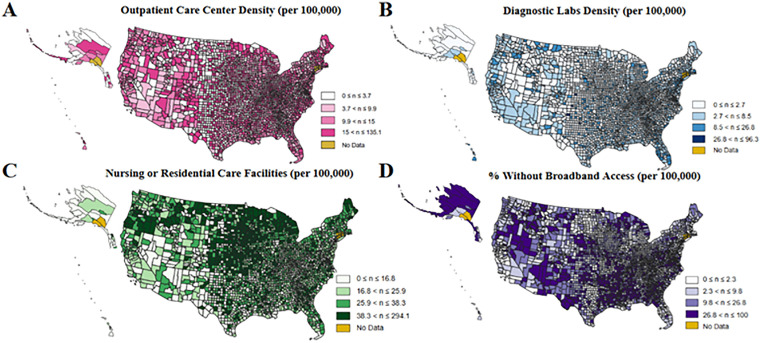
Geographical distributions of health facilities and sociodemographic characteristics. A. Density of outpatient care centers per 100,000 people. B. Density of diagnostic labs per 100,000 people. C. Density of nursing and residential care facilities per 100,000 people. D. Percentage of households without broadband access. Map base layer was obtained from the U.S. Census Bureau TIGER/Line Shapefiles, which are in the public domain [14].

Using heatmaps to visually represent the county-level geographical distributions of outpatient care centers, diagnostic labs, and nursing and residential care facilities across the United States, our analysis revealed a high density of all three types of facilities in the West Coast and New England regions of the US. We observed elevated densities of diagnostic labs in the South and of nursing facilities in the South and Midwest, suggesting regional variations in resource distribution. Notably, all three types of facilities exhibited a significant dearth in a vertical belt across the central United States that traverses the left of the Great Plains region and continues into the Southwest region (Fig 2A-2C). In addition to facility distributions, we visualized the percentage of households without broadband access. Consistent with the spatial patterns observed for healthcare facilities, we observed a distinct belt across the Midwest characterized by a higher percentage of households lacking broadband access (**Fig 2D**).

#### *Regression analysis*.

After adjusting for sociodemographic covariates, multivariable linear regression demonstrated that a 1% increase in the percentage of a county’s population without broadband access is linked to a decrease in outpatient care centers (β = -0.045, P < 0.001), diagnostic labs (β = -0.024, P < 0.001), and nursing and residential care facilities (β = -0.089, P < 0.001) per 100,000 people. Though broadband access was consistently associated with reduced health county-level health facility density across all types, the relationships between other sociodemographic factors and facility availability varied by facility type. With respect to race, a 1% increase in the percent of a county’s population identifying as White was associated with fewer diagnostic laboratories per 100,000 residents (β = -0.11; P = 0.0012). No statistically significant associations were observed for outpatient care centers (P = 0.25) or nursing and residential care facilities (P = 0.32). With respect to urbanicity, counties with a higher (more rural) NHCS urban-rural classification code had significantly fewer diagnostic laboratories (β = -0.23; P < 0.001), but significantly more outpatient care centers (β = 0.80; P < 0.001) and nursing and residential care facilities (β = 4.8; P < 0.001). With respect to poverty, a 1% increase in the percentage of households living below the poverty line was linked to an increase of 0.25 outpatient care centers per 100,000 residents (P < 0.001), but was not significantly associated with diagnostic labs (P = 0.31) or nursing and residential care facilities (P = 0.79) (**Table 2**).

**Table 2:** The effect of broadband access, urbanization status, household poverty rate, and race on the population density of outpatient care centers, diagnostic labs, and nursing/residential facilities per 100,000 residents (two-sided *t*-test). The adjusted multivariable linear regressions controlled for the following sociodemographic covariates: urban-rural status, percentage of households living in poverty, employment rate, insurance rate, sex, age, and race, as defined in the Methods section. Urbanization status was defined using the 2013 National Center for Health Statistics (NCHS) Urban–Rural Classification Scheme, which categorizes counties as: 1 = Large Central Metro (reference), 2 = Large Fringe Metro, 3 = Medium Metro, 4 = Small Metro, 5 = Micropolitan, and 6 = Noncore.

	Covariate	Unadjusted Analysis				Adjusted Analysis			
		β	95% CI (Low)	95% CI (High)	*P* value	β	95% CI (Low)	95% CI (High)	*P value*
**Outpatient Care Centers**	**Percent without Broadband**	-0.040	-0.056	-0.024	P < 0.001	-0.045	-0.063	-0.027	P < 0.001
	**Urbanization Status**	0.03845	-0.20	0.27	0.748	0.80	0.51	1.08	P < 0.001
	**Household Poverty Rate**	0.16	0.091	0.23	P < 0.001	0.25	0.13	0.37	P < 0.001
	**Percent of Individuals Identifying as White**	-0.055	-0.075	-0.034	P < 0.001	-0.089	-0.24	0.064	0.25
**Diagnostic Labs**	**Percent without Broadband**	-0.040	-0.047	-0.032	P < 0.001	-0.024	-0.032	-0.015	P < 0.001
	**Urbanization Status**	-0.64	-0.75	-0.54	P < 0.001	-0.23	-0.36	-0.099	P < 0.001
	**Household Poverty Rate**	-0.027	-0.058	0.0048	0.097	0.028	-0.02545034	0.081	0.31
	**Percent of Individuals Identifying as White**	-0.027	-0.036	-0.018	P < 0.001	-0.11	-0.18	-0.045	0.0012
**Nursing/Residential Care Facilities**	**Percent without Broadband**	-0.042	-0.078	-0.0065	0.021	-0.089	-0.13	-0.050	P < 0.001
	**Urbanization Status**	3.56	3.05	4.07	P < 0.001	4.8	4.2	5.4	P < 0.001
	**Household Poverty Rate**	-0.50	-0.65	-0.35	P < 0.001	0.034	-0.21	0.28	0.79
	**Percent of Individuals Identifying as White**	0.26	0.21	0.30	P < 0.001	-0.16	-0.49	0.16	0.32

#### Sensitivity analysis.

The findings of the descriptive analysis in Table 1 were further validated by the results of our sensitivity analysis, wherein high and low broadband access were defined using more stringent cutoffs based on the top and bottom quartiles of broadband access. Notably, we observed more pronounced effect sizes in the sensitivity analysis, suggesting that disparities may be concentrated in the most underserved counties. Moreover, the quartile-based comparison revealed significant differences in racial composition, with “low broadband access” having a lower percentage of White residents (75.30% vs. 78.38%; P = 0.001) (**S1 Table**).

## Discussion

Telemedicine has long been touted as a solution to rural-urban and socioeconomic disparities. However, in an era characterized by many post-pandemic telehealth interventions, rural communities with inadequate physical health facility presence may not benefit equitably from broadband internet-dependent telehealth services. Our results show a strong link between low broadband access and reduced availability of outpatient care centers and diagnostic labs, suggesting that rural and low-income communities may experience dual-disparities in access to both telehealth and in-person care.

Our findings align with previous studies that focus on the numerous sociodemographic disparities in broadband internet access. For example, previous studies have shown that rural populations generally face significant challenges in accessing specialty healthcare services. Due to the lack of specialists in rural areas, there is a greater reliance on primary care providers for conditions that often require specialized care which can delay early disease detection and complicate chronic disease management [16]. Often, people living in rural areas face long distances to healthcare facilities, higher out-of-pocket healthcare costs and limited insurance coverage, and the lack of critical healthcare infrastructure [17]. However, there has been limited focus on how broadband access may correspond with the distribution of these facilities. This study expands on the literature by examining the relationship between broadband internet access and the availability of various US outpatient care across the care continuum—including outpatient care centers, diagnostic labs, and nursing/residential care facilities. These facilities are critical because they provide essential services like early disease detection, diagnostic testing, and long-term care for chronic conditions—all of which are essential in ensuring continuous and comprehensive care. These findings shed light on the digital divide’s community-level impact on physical healthcare infrastructure, particularly in underserved rural and low-income communities, and highlights critical geographical and socioeconomic dual-disparities.

The connection between limited broadband access and reduced availability of healthcare facilities prompts the question of whether these services face lower demand in under-resourced areas. However, previous studies have shown that these areas actually have a higher demand for these services. In one study, people from rural communities often have a higher demand for healthcare services due to a greater burden of chronic diseases, such as diabetes, heart disease, and obesity. In addition, the study found that rural areas had significantly higher mortality rates from these chronic diseases compared to urban areas, including 193.5 vs. 161.7 deaths per 100,000 from heart disease, 180.4 vs. 158.3 from cancer, and 54.3 vs. 38.9 from chronic lower respiratory disease [18]. Our results are especially concerning because these populations face disproportionate health challenges, yet have reduced access to both broadband internet and in-person outpatient facilities. Although the absolute difference in outpatient care facility density between “low” and “high” broadband access counties we present in this study (1.45 centers per 100,00 residents) may appear modest, even small disparities can have meaningful implications for patient access in rural and underserved counties—where a single facility may serve a large catchment area [19]. In such counties, the absence of a single facility may delay care, increasing the likelihood of advanced disease progression and strain on overburdened emergency departments. When scaled across counties nationwide, these seemingly small per-capita differences may reflect substantial disparities in care availability at the population level.

### Recommendations

To promote telehealth as a viable solution to combat rural health disparities, comprehensive community, clinical, and federal-level interventions, targeting both increased broadband access and physical health facility expansion, must be enacted. In the short term, initiatives to promote the development of broadband infrastructure may reduce disparities in healthcare access in rural communities. The FCC’s Affordable Connectivity Program (ACP) provided discounts on internet services and electronic devices to eligible households, with special provisions for households on Tribal lands. The ACP provided internet access to 21 million people, half of whom reported no or solely mobile internet service prior to their ACP benefit, with 80% reporting that affordability was the primary concern in having inconsistent or no service [20]. However, the ACP was discontinued in June 2024 due to a lack of congressional funding. One effective immediate intervention would be for Congress to reinstate funding for the ACP to support households that relied on it for broadband access, which were disproportionately low-income and Tribal households, including 15% from rural areas [21]. In tandem, given the underutilization of the ACP by eligible families during its implementation, it is important to raise awareness of programs like Link Health, which assists families in signing up for the ACP [22].

Aside from publicly funded programs, eligibility and benefit expansion of private sector initiatives, such as T Mobile’s Project 10 Million can expand broadband access to underserved communities [23]. Project 10 Million aims to provide students qualifying for the National School Lunch Program with free internet and mobile hotspots and access to low-cost electronic devices. Expanding Project 10 Million provisions to geographic areas lacking in health facilities can assist families with accessing telehealth services, potentially bridging both health and educational disparities. Additional policies for improved telehealth access among rural and low-income communities include increased screening services, broadband infrastructure expansion, mandatory compensation for financial losses incurred during screening visits, and targeted investment in additional health facilities in high-burden regions, all of which are necessary to bridge the gap in access to early preventative care.

Furthermore, it is critical to address the shortage of in-person medical care in rural areas with limited access to telehealth services. Such efforts include increasing funding for rural health clinics to expand their staffing capacity, offering loan repayment and financial incentives to healthcare providers who commit to serving in rural areas, and implementing mobile health units to deliver essential medical services to remote regions. The MOBILE Health Care Act passed in 2023 expanded the development of mobile clinics through the use of federal funds, resulting in a 40 percent growth in mobile health center units since 2019 [24]. Sustained and increased funding for this program can continue to incentivize the development of mobile health clinics for rural communities, increasing healthcare access. Additionally, policies should focus on expanding residency programs in rural hospitals, supporting the integration of telehealth into existing healthcare infrastructure, and creating partnerships between urban and rural healthcare systems to facilitate resource sharing and provider rotations.

### Limitations

This study bears several limitations. While providing valuable insights into broadband access and health facility density in US counties, the data does not include broadband service quality or post-2020 broadband accessibility data and thus may not fully capture post-COVID developments in infrastructure. Moreover, although we explore population-level demographic characteristics that may influence the need for health facilities across counties, our data does not explicitly quantify differences in need; thus, future research should aim to elucidate such factors that may influence facility distribution. Furthermore, the reliance on county-level data may mask neighborhood-level variations and disparities, highlighting the necessity for a more granular investigation into factors influencing healthcare access in rural areas. Furthermore, our data does not consider broadband speeds, which remain an influential measure of the quality of broadband services beyond accessibility. Finally, our study does not assess the relationship between broadband access and the density of emergency departments, which play a crucial role in providing immediate healthcare services, especially in rural or underserved areas.

## Conclusions

Our research highlights a link between broadband inadequacies and scarcity of healthcare facilities in rural communities, underscoring the need for targeted broadband expansion in these areas as a critical strategy to enhance telehealth services and, by extension, improve healthcare access and outcomes. By investing in broadband infrastructure and attracting and retaining physicians in rural communities, policymakers can facilitate more effective delivery of telehealth services and address the root causes of healthcare disparities that disproportionately affect rural populations, ensuring that rural communities are not left behind in the digital age.

## Supporting information

S1 Table Sensitivity analysis of U.S. county-level baseline characteristics and health facility density per 100,000 residents, stratified by broadband internet access defined using national quartiles of the percentage of households without broadband access. Counties with high broadband access were defined as those in the lowest national quartile (≤ 2.3%), and counties with low broadband access were defined as those in the highest national quartile (≥ 26.8%) (two-sided t-test).
(DOCX)

S1 Fig Geographic regions of the United States, included to facilitate the interpretation of qualitative geographical patterns described in the Results section. Map base layer was obtained from the U.S. Census Bureau TIGER/Line Shapefiles, which are in the public domain [14].
(TIF)

S1 Data United States county-level dataset containing county identifiers, population, broadband access, healthcare facility density measures, urbanicity, and sociodemographic variables used in the analyses. (CSV)
