# Supplementary material for: Healthcare unplugged: Disparities in broadband internet and health facility access among US counties
Source: PLOS Digit Health. 2026 Jul 23;5(7):e0000732. doi: 10.1371/journal.pdig.0000732 (PMC13395355; doi:10.1371/journal.pdig.0000732)
Supplement: S1 Table — Counties with high broadband access were defined as those in the lowest national quartile (≤ 2.3%), and counties with low broadband access were defined as those in the highest national quartile (≥ 26.8%) (two-sided t-test). (DOCX) [file pdig.0000732.s001.docx]

**S1 Table:** Sensitivity analysis of U.S. county-level baseline characteristics and health facility density per 100,000 residents, stratified by broadband internet access defined using national quartiles of the percentage of households without broadband access. Counties with high broadband access were defined as those in the lowest national quartile (≤ 2.3%), and counties with low broadband access were defined as those in the highest national quartile (≥ 26.8%) (two-sided t-test).

|  | **Density (per 100,000 residents)** | | | ***P* value (two-sided *t* test)** |
| --- | --- | --- | --- | --- |
|  | **All US Counties** | **US counties with low broadband access** | **US counties with high broadband access** |  |
| **NHCS Urban-Rural Code** | 4.64 | 5.42 | 3.86 | P < 0.001 |
| **Household Poverty Rate** | 10.17 | 12.21 | 8.88 | P < 0.001 |
| **Age** | 41.60 | 43.18 | 40.52 | P < 0.001 |
| **Percent of Population Identifying as White** | 79.05 | 75.30 | 78.38 | 0.0011 |
| **Percent of Adults With a High School Degree** | 88.34 | 86.28 | 89.23 | P < 0.001 |
| **Percent of Adults Who are Employed** | 55.12 | 50.57 | 58.35 | P < 0.001 |
| **Percent of Adults With Insurance** | 90.48 | 88.76 | 91.38 | P < 0.001 |
| **Outpatient Care Centers** | 11.18 | 9.87 | 11.95 | P < 0.001 |
| **Diagnostic Labs** | 2.93 | 1.59 | 4.07 | P < 0.001 |
| **Nursing/Residential Facilities** | 31.01 | 30.32 | 30.60 | 0.81 |
