# Supplementary figures and images for: Healthcare unplugged: Disparities in broadband internet and health facility access among US counties
Source: PLOS Digit Health. 2026 Jul 23;5(7):e0000732. doi: 10.1371/journal.pdig.0000732 (PMC13395355; doi:10.1371/journal.pdig.0000732)

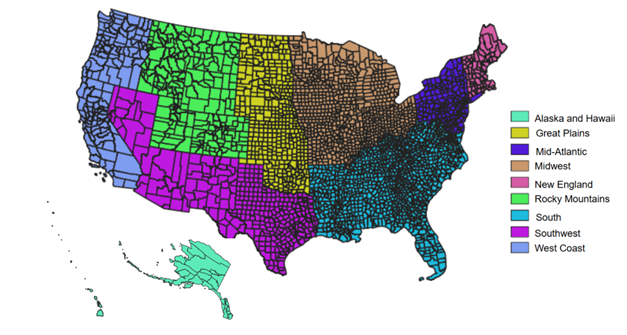

Supplement: S1 Fig — Map base layer was obtained from the U.S. Census Bureau TIGER/Line Shapefiles, which are in the public domain [14]. (TIF) [file pdig.0000732.s002.tif]
